# Expression Profile of Six RNA-Binding Proteins in Pulmonary Sarcoidosis

**DOI:** 10.1371/journal.pone.0161669

**Published:** 2016-08-30

**Authors:** Zdenka Navratilova, Eva Novosadova, Michael Hagemann-Jensen, Susanna Kullberg, Vitezslav Kolek, Johan Grunewald, Martin Petrek

**Affiliations:** 1 Laboratory of Immunogenomics and Immunoproteomics, Department of Pathological Physiology, Faculty of Medicine and Dentistry Palacky University, Olomouc, Czech Republic; 2 Respiratory Medicine Unit, Department of Medicine, Karolinska Institutet, Stockholm, Sweden; 3 Institute of Molecular and Translational Medicine, Faculty of Medicine and Dentistry, Palacky University, Olomouc, Czech Republic; 4 Respiratory Medicine Unit, Department of Medicine, Solna & Center for Molecular Medicine, Karolinska Institutet, Stockholm, Sweden; 5 Department of Respiratory Medicine, Palacky University, Olomouc, Czech Republic; Colorado State University, UNITED STATES

## Abstract

**Background:**

Sarcoidosis is characterised by up-regulation of cytokines and chemokine ligands/receptors and proteolytic enzymes. This pro-inflammatory profile is regulated post-transcriptionally by RNA-binding proteins (RBPs). We investigated *in vivo* expression of six RBPs (AUF1, HuR, NCL, TIA, TIAR, PCBP2) and two inhibitors of proteolytic enzymes (RECK, PTEN) in pulmonary sarcoidosis and compared it to the expression in four control groups of healthy individuals and patients with other respiratory diseases: chronic obstructive pulmonary disease (COPD), asthma and idiopathic interstitial pneumonias (IIPs).

**Methods:**

RT-PCR was used to quantify the mRNAs in bronchoalveolar (BA) cells obtained from 50 sarcoidosis patients, 23 healthy controls, 30 COPD, 19 asthmatic and 19 IIPs patients. Flow cytometry was used to assess intracellular protein expression of AUF1 and HuR in peripheral blood T lymphocytes (PBTLs) obtained from 9 sarcoidosis patients and 6 healthy controls.

**Results:**

Taking the stringent conditions for multiple comparisons into consideration, we consistently observed in the primary analysis including all patients regardless of smoking status as well as in the subsequent sub-analysis limited for never smokers that the BA mRNA expression of AUF1 (*p*<0.001), TIA (*p*<0.001), NCL (*p*<0.01) and RECK (*p*<0.05) was decreased in sarcoidosis compared to healthy controls. TIA mRNA was also decreased in sarcoidosis compared to both obstructive pulmonary diseases (COPD and asthma; *p*<0.001) but not compared to IIPs. There were several positive correlations between RECK mRNA and RBP mRNAs in BA cells. Also sarcoidosis CD3+, CD4+ and CD8+ PBTLs displayed lower mean fluorescence intensity of AUF1 (*p≤*0.02) and HuR (*p≤*0.03) proteins than control healthy PBTLs.

**Conclusion:**

mRNA expressions of three RBPs (AUF1, TIA and NCL) and their potential target mRNA encoding RECK in BA cells and additionally protein expression of AUF1 and HuR in PBTLs were down-regulated in our sarcoidosis patients compared to healthy individuals. Its significance, e.g. for stability of mRNAs encoding pro-inflammatory factors, should be further explored in sarcoidosis.

## Introduction

Pulmonary sarcoidosis is characterised by parenchymal granulomas of unknown cause(s) [1, 2]. A typically observed immunological feature is a helper T cell type 1 (Th1) polarisation of chronic inflammation with elevated secretion of interleukin (IL)-2, IL-8, IL-12, interferon (IFN) gamma and tumour necrosis factor (TNF) alpha at sites of this disease. A contribution of Th2 cytokine profile (e.g. IL-4 and IL-6) is hypothesized in advanced sarcoidosis[1, 3]. Furthermore, there is an increasing biosynthesis of pro-fibrogenic factors (e.g. tumour growth factor (TGF) beta) and proteolytic enzymes (e.g. matrix metalloproteinases (MMP)) during sarcoidosis progression [1, 2, 4–6].

Besides sarcoidosis[4, 5], other respiratory diseases, including chronic obstructive pulmonary disease (COPD), asthma, and idiopathic interstitial pneumonia (IIPs), are associated with a similar elevation of some pro-inflammatory factors and MMPs (e.g.MMP-9) [7–9].

Inflammation, polarization of Th immune responses and expression of the corresponding pro-inflammatory factors are modulated at post-transcriptional level[10–13]. In this process, two components are of great importance for the post-transcriptional regulation: RNA-binding proteins (RBPs) and microRNAs. Contrary to microRNA’s specific binding sites, RBPs bind AU-rich elements (ARE) that relatively commonly exists at 3’ end of mRNA, encoding for several cytokines, transcriptional factors and MMPs[10–12]. Subsequently, the RBP-ARE interactions modulate the fate of the targeted mRNA. Among various RBPs, HuR (alias ELAVL1) usually stabilizes the targeted mRNA while the inhibitory RBPs such as AUF1 (alias HNRNPD) and TIA/TIAR complex act against the stability and translation, respectively [10–12].

In particular, HuR stabilizes Th2 transcription factor GATA3 mRNA as well as Th2 specific cytokine mRNAs of IL-4 and IL-13 and thus promotes a polarisation of immune response to Th2 [14]. Another RBP called AUF1 promotes the mRNA destabilization of IL-6, but can also support mRNA expression under specific conditions[15, 16]. The function of the active RBPs is usually associated with their cytoplasmic accumulation, but they also shuttle to nucleus where they can first meet their target mRNA sequence[17]. In the context of sub-cellular localisation, another RBP called NCL (alias nucleolin) has an unique property as it is additionally present on the cellular surface of macrophages where it mediates phagocytosis of apoptotic cells[18]. Of these RBPs, NCL, HuR and AUF1 have all been reported to affect MMP-9 expression[19–21]

A lot of potential targets of the RBPs, including MMP-9, have already been reported to be dysregulated in sarcoidosis [1, 2, 4, 5, 10, 19–22]. To our knowledge, no clinical study has yet addressed the *in vivo* expression of RBPs in pulmonary sarcoidosis or other non-malignant pulmonary pathologies. Two inhibitors of MMP-9 termed RECK (reversion-inducing-cysteine-rich protein with kazal motifs) and PTEN (phosphatase and tensin homolog) also have AU-rich elements (www.AREsite.com) [23–25] but limited information about their expression exists in lung diseases.

We, therefore, decided to evaluate *in vivo* bronchoalveolar (BA) expression of six RBPs (AUF1, HuR, NCL, TIA, TIAR and PCBP2) and of two possible targets of RBPs (MMP-9 inhibitor RECK and PTEN) in our patients with pulmonary sarcoidosis and compare it with that in four control groups including healthy subjects, obstructive (COPD and asthma) and non-obstructive pathologies (IIPs).

## Methods

### Subjects

Bronchoalveolar lavage (BAL) was performed according to our standard protocol (Petrek et al 1993) from 50 patients with pulmonary sarcoidosis (male/female 25/25; mean age 44 years, min-max 21–77 years), 23 healthy control subjects (17/6; 43, 19–78), 30 COPD patients (18/12; 64, 40–84 years), 19 asthmatic patients (5/14; 45, 20–83) and 19 IIPs patients (6/13; 57, 33–80) (S1 File). BA cellular profiles of all study groups are provided in S1 Table. In addition, 9 patients with pulmonary sarcoidosis (men/women 5/4; mean age 54, min-max 41–80) and 6 healthy controls (1/5; mean age 45, min-max 35–59) provided peripheral blood samples for the analyses by flow cytometry.

Diagnosis of pulmonary sarcoidosis was made according to the criteria of ATS/ERS/WASOG International Consensus Statement[1]. The BAL samples were obtained from Czech pulmonary sarcoid patients without Löfgren‘s syndrome (n = 50) who were classified with CXR (chest X ray) stage I (n = 25) and CXR stage II (n = 25). Blood samples were obtained from Swedish pulmonary sarcoid patients with/without Löfgren‘s syndrome (n = 2/7) who were classified with CXR stage I (n = 1), II (n = 3), II-III (n = 1), III (n = 2) and IV (n = 2). COPD and asthma were defined according to the criteria of the Global initiative for chronic Obstructive Lung Disease (GOLD) [26]and Global INitiative for Astma (GINA)[27], respectively. All COPD patients had mild or moderate airway obstruction defined as an FEV1/FVC (Forced Expiratory Volume in one second / Forced Vital Capacity) ratio <0.7 and FEV1% predicted 50–79%. We futher enrolled the patients with IIPs based on typical clinical and radiological features together with the histopathological confirmation on surgical lung biopsy [28–30].

All samples of BAL were obtained in Department of Respiratory Medicine, Palacký University Hospital in Olomouc, the Czech Republic. Blood sampling (from different patients) was performed at the Karolinska Institutet in Stockholm, Sweden. All Czech and Swedish patients gave their informed consent to participate in the study, which was approved by the local Ethical committees of the Medical Faculty PU & University Hospital (Olomouc, the Czech Republic) and Karolinska Institutet (Stockholm, Sweden).

### BA cells processing, RNA isolation and reverse transcription

BA cells were separated from the fluid by centrifugation as described previously [31]. The total RNA was isolated with High Pure miRNA Kit (Roche, Germany). Reverse transcription was performed by Transcriptor First Strand cDNA Synthesis Kit (Roche, Germany).

### Gene expression measurements by real-time RT-PCR

RotorGene3000 system (Corbett Research, Sydney, Australia) was used to assess relative expression. RT-PCR reaction conditions and a reference gene are described elsewhere [32]. Full names of here measured genes with their general effect on inflammation, the corresponding primer sequences, and probes for the investigated genes are listed in S2 Table.

### PBMCs processing and flow cytometry

Heparinized whole blood was kept at room temperature. Peripheral blood mononuclear cells (PBMCs) were separated by Ficoll-gradient (Ficoll Paque PLUS, GE Healthcare, Uppsala, Sweden) and washed twice with cell wash (PBS, 0.5%BSA and 0.02%NaN_3_). From each sample, 0.5×10^6^ PBMCs were fixed by Fixation/Permeabilization Concentrate and Diluent (eBioscience), permeabilized by Permeabilization Buffer (eBioscience) and stained for surface proteins using the following antibodies; CD3-Pacific Blue (BD Pharmingen), anti CD4-APC-H7 (BD Pharmingen) and CD8-AmCyan (BD Pharmingen). For intracellular staining of cytoplasmic and nuclear proteins the following antibodies were used: polyclonal rabbit IgG anti-AUF1-APC (LifeSpan BioSciences), monoclonal mouse IgG1 kappa anti-HuR-APC (LifeSpan BioSciences), APC mouse IgG1 kappa isotype control (BD Pharmingen) and APC rabbit IgG isotype control (Santa Cruz biotechnology). Results are expressed as mean fluorescence intensity (MFI) minus background provided by the isotype-matched negative control antibodies. Samples were run on an eight-colour FACSCanto II flow cytometer (Becton Dickinson). Data were analysed with Flowjo 10, Treestar.

### Statistics

Mann-Whitney U-test was used to detect possible effect of cigarette smoking and aging in each disease (pulmonary S, COPD and asthma) and healthy control group. Kruskal-Wallis test with Dunn’s multiple comparison tested differences in the expression of the investigated genes among sarcoidosis, healthy controls and three patient control groups with COPD, asthma and IIPs (GraphPad Prism; GraphPad, La Jolla, CA USA). The primary analysis of Kruskal-Wallis test with Dunn’s multiple comparison included all recruited patients and the subsequent sub-analysis took into consideration smoking status. Correlations were examined using Spearman’s rank correlation coefficient (SPSS 12.0 for Windows; SPSS, Chicago, IL, USA). A *p* value < 0.05 was considered to be significant.

## Results

### Bronchoalveolar (BA) expression of RBP mRNA in pulmonary sarcoidosis compared to healthy controls

**Fig 1:**
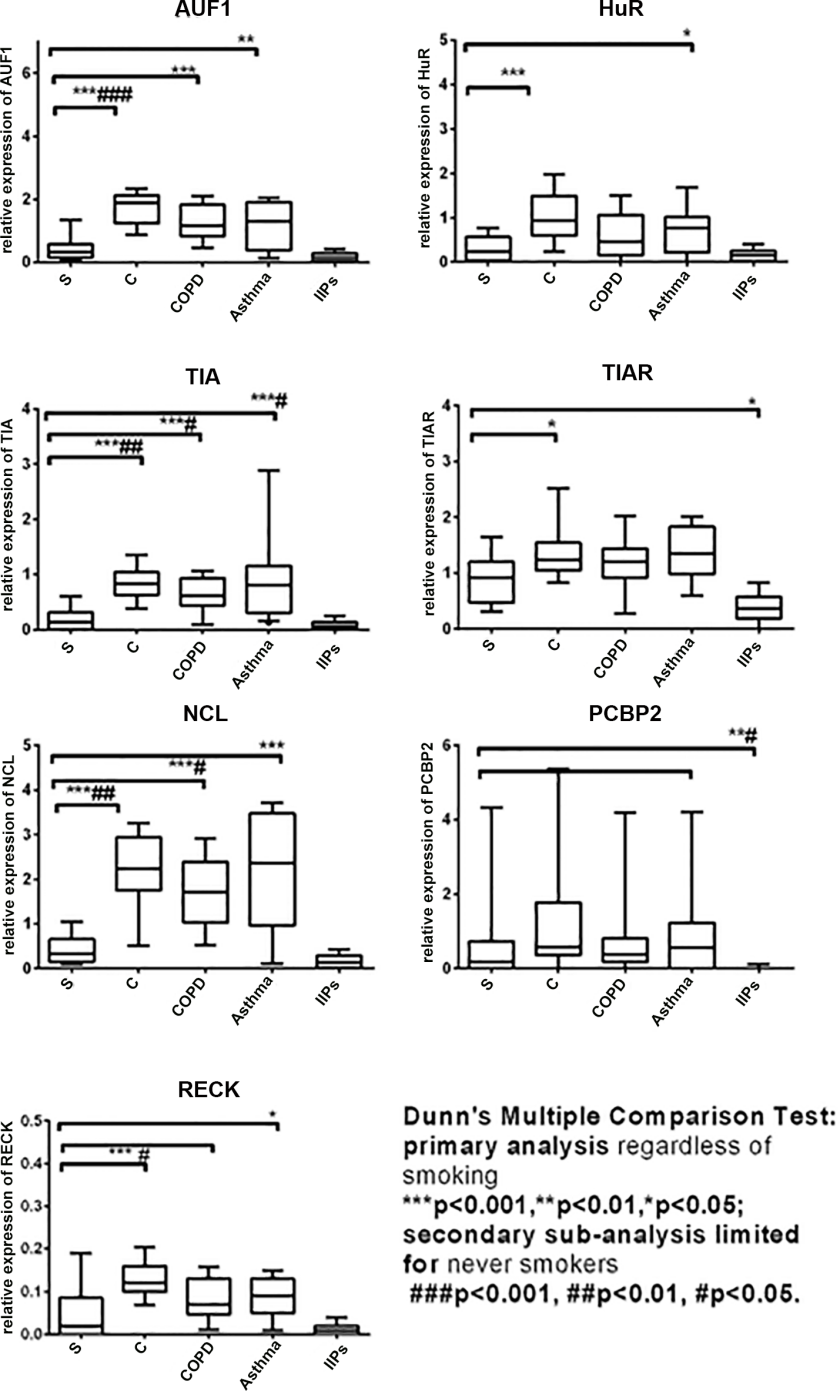
The relative mRNA expression of RNA-binding proteins (RBPs) and an inhibitor of proteolytic activity RECK in pulmonary sarcoidosis (S) in comparison with healthy controls (C) and patients with chronic obstructive pulmonary disease (COPD), asthma and idiopathic interstitial pneumonia (IIPs).

Dunn's Multiple Comparison Test of five investigated groups including all recruited patients regardless of smoking status showed significantly decreased relative expressions of AUF1 (*p*<0.001), HuR (*p*<0.001), TIA (*p*<0.001), TIAR (*p*<0.05) and NCL (*p*<0.001) and RECK (*p*<0.001) in BA cells from the patients with pulmonary sarcoidosis compared to those in healthy controls (Fig 1 and S3 Table). The relative expression of PTEN did not differ in the patients with pulmonary sarcoidosis compared to that in healthy controls. There was no difference in neither RBPs nor the inhibitors of MMPs between CXR (chest X ray) stage I and stage II (for all *p*>0.05). The subsequent sub-analysis of never smokers showed that AUF1 (*p*<0.001), TIA (*p*<0.001), NCL (*p*<0.01) and RECK (*p*<0.05) remained to be down-regulated in sarcoidosis compared to healthy controls (Fig 1 and S3 Table).

### Bronchoalveolar (BA) expression of RBP mRNA in pulmonary sarcoidosis compared to other control groups with COPD, asthma and IIPs

Regardless of smoking status, a decreased expression of AUF1 (*p*_*COPD*_<0.001, *p*_*asthma*_<0.01), TIA (*p*_*COPD*_<0.001, *p*_*asthma*_<0.001) and NCL (*p*_*COPD*_<0.001, *p*_*asthma*_<0.001) was observed in patients with sarcoidosis compared to both obstructive pathologies (COPD and asthma; Fig 1 and S3 Table). The subsequent sub-analysis of never smokers showed the decreased expression of TIA (*p*_*COPD*_<0.05, *p*_*asthma*_<0.05) in sarcoidosis patients compared to both obstructive pathologies (COPD and asthma) and additionally NCL (*p*_*COPD*_<0.05) compared to COPD patients (Fig 1 and S3 Table).

### Bronchoalveolar (BA) expression of RBP mRNA in other pulmonary diseases

Comparisons among 4 control groups of healthy controls and disease controls (COPD, asthma and IIPs) are summarised in supplement and S4 Table. Briefly, AUF1 (*p*<0.001), TIA (*p*<0.001), NCL (*p*<0.001), PCBP2 (*p*<0.001) and RECK (*p*<0.001) were down-regulated in IIPs compared to healthy controls.

### The effect of smoking and age on BA expression of RBP mRNA

The possible effect of smoking was investigated by several analyses between never smokers and smokers (S1 File). The expression of RBPs and two inhibitors of proteolytic enzymes did not differ between the sub-groups of our patients with pulmonary sarcoidosis (*p*>0.05).

Regarding age, the relative expressions did not differ between our young (*≤*45 years, n = 25) and elderly (>45 years, n = 25) patients with pulmonary sarcoidosis (*p*>0.05). The data on the effect of age in control groups is provided in S1 File.

### Correlation analyses

To investigate *in vivo* relationship between RBPs and the possible targets, we performed several correlation analyses between the BA mRNA expressions of RBPs and the BA mRNA expressions of genes encoding for inhibitors of MMPs with ARE. Among sarcoidosis patients, RECK decreased in parallel with decreasing expression of AUF1 (*p* = 0.002), NCL (*p* = 0.02), HuR (*p* = 0.002), TIA (*p*<0.001) and TIAR (*p* = 0.007). There was no relationship between PTEN and RBPs in our patients with sarcoidosis.

Regarding cellular profile of BAL (bronchoalveolar lavage), the BA relative expression of PCBP2 mRNA showed negative correlations with absolute and relative numbers of lymphocytes (*p* = 0.003 and *p* = 0.003) in the patient group of pulmonary sarcoidosis. The information on other relationships between the cellular profile of BAL and mRNA expressions in all patient groups is provided in S1 File.

### RBPs in peripheral blood mononuclear cells (PBMCs) obtained from patients with pulmonary sarcoidosis

**Fig 2:**
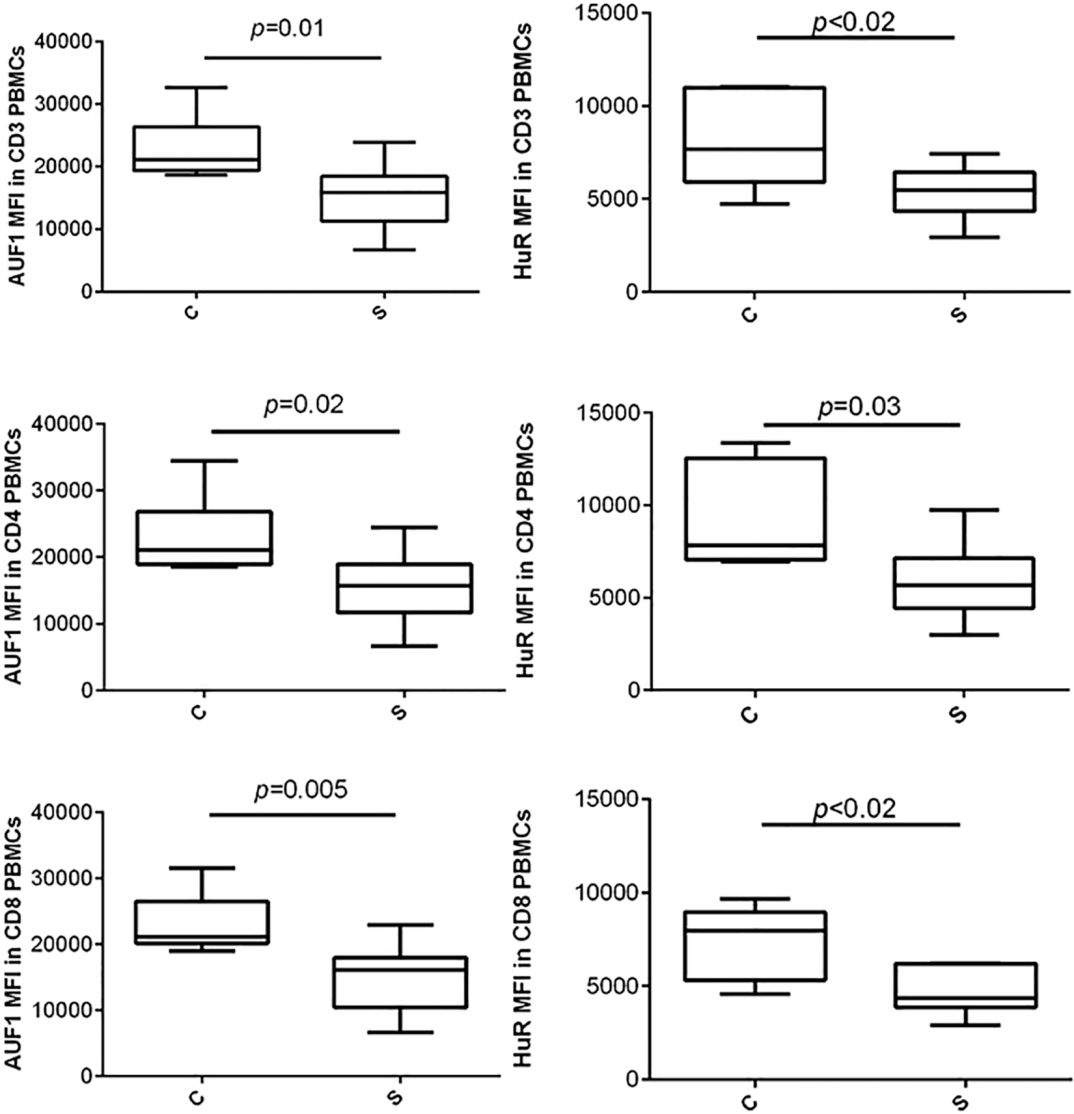
AUF1 and HuR protein expressions in CD3+, CD4+ and CD8+ T lymphocytes of peripheral blood obtained from the patients with pulmonary sarcoidosis and healthy controls. Legend: PBMC, peripheral blood mononuclear cells; MFI, mean fluorescence intensity. The data are expressed as whisker box plots; the box represents the 25–75th percentiles, the median is indicated by a bar across the box, the whiskers on each box represent the 10–90th percentiles.

Because the investigation of RBPs in sarcoidosis was our primary aim, two of the most extensively studied RBPs (AUF1 and HuR) were further selected for the investigation of RBP protein expression in sarcoidosis peripheral blood T lymphocytes (PBTLs). Total CD3+ as well as CD4+ and CD8+ PBTLs obtained from PBMCs of the patients with sarcoidosis displayed lower mean fluorescence intensity (MFI) of AUF1 (*p≤*0.02) and HuR (*p≤*0.03) than those from healthy controls (Fig 2).

## Discussion

There has been yet no information on *in vivo* expression of RNA-binding proteins (RBPs) in respiratory diseases. Taking the stringent conditions for multiple comparisons and smoking status into consideration, we observed the decreased expression of three RBPs (AUF1, TIA and NCL) in bronchoalveolar (BA) cells from our patients with pulmonary sarcoidosis compared to those from our healthy controls. Additionally, TIA was also down-regulated in both airway obstructive pathologies (COPD and asthma) but not in our patients with another diffuse lung disease, IIP. Regarding inhibitors of proteolytic enzymes being under post-transcriptional control of RBPs, lower expression of RECK was observed in BA cells in all investigated patient groups compared to healthy controls. Furthermore, several RBPs correlated with BA expression of RECK in different lung inflammatory pathologies. Although these correlations provide only an indirect proof of physical contact between RBPs and RECK, their *in vivo* relationship is likely.

The strength of our work lies within the *in vivo* assessment of clinically relevant biological material such as BA cells that are in tight contact with the lung inflammatory processes. In this study, RBPs were not affected by either cigarette smoking or age. In line with our data on smoking, Hudy et al reported that cigarette smoke extract does not induce any dysregulation of RBPs (AUF1 and HuR) in primary human bronchial epithelial cells [33]. In contrast to these RBPs, Glader et al. observed that *in vitro* stimulation by cigarette smoke extract reduces the expression of another RBP (TIA) [34]. Because of the inconsistent *in vitro* [33, 34] and our *in vivo* data on TIA, we have performed both primary analysis regardless smoking status and then the smoking status-matched case-control sub-analyses. The case-control sub-analysis of never smokers with sarcoidosis showed that the down-regulation of AUF1, TIA and NCL in our patients with sarcoidosis is independent of smoking status. By contrast, the down-regulation of HuR and TIAR in sarcoidosis did not reach significant level in the sub-analysis of never smokers. The inconsistent results between our primary analyses of all patients and our subsequent sub-analyses of never smokers suggest that the expression of HuR and TIAR could be partly affected by the smoking status but did not reach significant level because the effect of smoking is not profound.

Similar to sarcoidosis, we observed several RBPs (AUF1, TIA, NCL and addition TIAR and PCBP2) to be decreased in our patients with IIPs, but not in our patients with airway obstructive pathologies (COPD and asthma) when comparing to healthy controls. Furthermore, the expression of TIA was additionally decreased in our patients with sarcoidosis compared to those with both airway obstructive pathologies (COPD and asthma) but not compared to those with another diffuse lung disease, IIP. The comparison with two obstructive pathologies suggests that AUF1, NCL and mainly TIA are important regulation factors namely in pulmonary interstitial lung diseases.

One may argue that the observed decreased expressions of RBPs (AUF1, NCL and TIA) may not be associated with sarcoidosis but rather be an effect of the elevated sub-population of BA lymphocytes being a hallmark of pulmonary sarcoidosis. In line with this hypothesis, we observed the correlation between PCBP2 mRNA and BA lymphocyte sub-population in our patients with pulmonary sarcoidosis. In contrast to PCBP2, the mRNA expression of other RBPs and two inhibitors of MMP-9 (RECK and PTEN) did not correlate with any BA sub-population in our patients with sarcoidosis. However, we can not exclude the possibility that the sarcoidosis down-regulation of some RBP mRNAs (e.g. TIA) is primarily dependent on other BA sub-populations that were not assessed in our patients with sarcoidosis, e.g. the reduced number of BA natural killer T cells in sarcoidosis [35]. Future studies should, therefore, directly assess the RBPs in the particular BA sub-populations including alveolar macrophages and broad spectrum of different T lymphocytes.

To start with the investigation of cell sub-populations, we further used flow cytometry to investigate the protein expressions of AUF1 and HuR in peripheral blood T lymphocytes (PBTLs) obtained from independent group of sarcoidosis patients. The expression of AUF1 protein was consistently down-regulated in both CD4+ and CD8+ PBTLs from our patients with sarcoidosis compared to those from healthy controls. Similar to the AUF1 protein, we observed the decreased expression of HuR protein in our patients with sarcoidosis.

In the healthy lung and in leukocytes, RBPs are more highly expressed than microRNAs and even than transcription factors, possibly indicating the importance of RBPs in monitoring of lung inflammation[36]. At present time, however, most human studies on RBPs are restricted to malignancies. Actually, as late as the last year (2014), a complex expression of RBPs was investigated in various cancers[36]. A general abundance of RBPs was reported in lung carcinoma. It is in contrast with the mRNA down-regulation of AUF1, NCL and TIA in BA cells and the protein down-regulation of AUF1 and HuR in PBTL from our patients with sarcoidosis. Based on current knowledge [4, 5, 15, 16, 19], we can only speculate that the down-regulated expressions may support sarcoid inflammation by an insufficient degradation of mRNA encoding for cytokines (e.g. IL-6), chemokines (e.g. IL-8) and proteolytic enzymes (e.g. MMP-9). In parallel, the effect of RBPs (e.g. HuR) on the pro-inflammatory factors may be mediated by targeting mRNA encoding for transcriptional factors and second messengers in specific sub-populations of differentiated T lymphocytes[37]. Genome-wide measurement of stead-state mRNA in the particular sub-populations of at least circulating T lymphocytes could therefore bring new information on post-transcriptional regulation mechanisms in sarcoidosis[38].

RECK and PTEN with their ARE sites are also potential targets of the RBPs-mediated regulation of inflammation.

RECK protein is a cellular membrane anchored glycoprotein that inhibits both expression and proteolytic activity of MMP-9 [24]. The low mRNA expression of RECK in our patients with different pulmonary pathologies could therefore support the well-known increased proteolytic degradation of connective tissue in these inflammatory lung diseases [39, 40]. In addition, the low mRNA expression of RECK in our asthmatic patients is in line with a previous observation on sputum samples from asthmatic patients [41].

We also observed the mRNA expression of RECK to correlate with five RBPs (AUF1, HuR, NCL, TIA and PCBP2). However, the particular interaction between RECK ARE site and RBP has not been experimentally investigated so far. Our correlation analysis also provides only indirect proof of physical interaction. We must therefore mention that the *in vivo* relationship may be also mediated by other RBPs (e.g. TTP) and other regulation mechanisms[12]. In this context, an important aspect is that we only measured mRNA expressions of BA RBPs whose protein concentrations do not have to correlate with the expression of the putatively targeted mRNA encoding for RECK gene. Other clinical studies should therefore assess RBPs and their potential targets at protein levels. Second, post-translation phosphorylation and sub-cellular localisation of RBPs may play important role in the post-transcriptional regulation of RECK mRNA stability and its translation [12]. Third, other important factor of post-transcription regulation is a silencing of targeted mRNA by microRNA [12, 22].

## Conclusion

The down-regulated mRNA expressions of three RBPs (AUF1, TIA and NCL) in unseparated bronchoalveolar cells and the down-regulated protein expressions of two RBPs (AUF1 and HuR) in peripheral blood T lymphocytes were observed in our patients with pulmonary sarcoidosis. These expression data indicate down-regulated expressions of the RBPs in sarcoidosis. Further, low mRNA expression of RECK was present in our patients with pulmonary sarcoidosis, COPD and IIPs. The low expression of RECK may shift a proteinase / anti-proteinase balance to excessive proteolytic character in these diseases. Further studies are required to clarify the particular targets that are regulated by RBPs in bronchoalveolar cells in diffuse and inflammatory lung disease. The assessment of stead-state mRNA encoding for the particular targeted gene is also desirable as it may bring key information on role of RBPs in the chronic inflammation of sarcoidosis and other respiratory diseases.

## Supporting Information

S1 File Supplement. (DOC)

S1 Table BA cellular profiles of investigated groups. (DOC)

S2 Table Primer sequences for RNA-binding proteins (RBPs), two inhibitors of proteolytic activity (RECK and PTEN) and a housekeeping gene (PSMB2). (DOC)

S3 Table mRNA expression for RNA-binding proteins (RBPs) and one inhibitor of proteolytic activity (RECK) in the patients with pulmonary sarcoidosis compared to 4 control groups including healthy individuals and the patients with COPD, asthma and IIPs. (DOC)

S4 Table Comparisons of mRNA expression for RNA-binding proteins (RBPs) and one inhibitor of proteolytic activity (RECK) between healthy controls and patient groups including chronic obstructive pulmonary disease (COPD), asthma and idiopathic interstitial pneumonias (IIPs). (DOC)

## References

[1] Statement on sarcoidosis. Joint Statement of the American Thoracic Society (ATS), the European Respiratory Society (ERS) and the World Association of Sarcoidosis and Other Granulomatous Disorders (WASOG) adopted by the ATS Board of Directors and by the ERS Executive Committee, February 1999. Am J Respir Crit Care Med. 1999;160(2):736–55. Epub 1999/08/03. 10.1164/ajrccm.160.2.ats4-99 . 10430755

[2] Valeyre D, Prasse A, Nunes H, Uzunhan Y, Brillet PY, Muller-Quernheim J. Sarcoidosis. Lancet. 2014;383(9923):1155–67. Epub 2013/10/05. 10.1016/S0140-6736(13)60680-7 . 24090799

[3] Patterson KC, Hogarth K, Husain AN, Sperling AI, Niewold TB. The clinical and immunologic features of pulmonary fibrosis in sarcoidosis. Transl Res. 2012;160(5):321–31. 10.1016/j.trsl.2012.03.005 22683422 PMC3910531

[4] Henry MT, McMahon K, Mackarel AJ, Prikk K, Sorsa T, Maisi P, et al Matrix metalloproteinases and tissue inhibitor of metalloproteinase-1 in sarcoidosis and IPF. The European respiratory journal: official journal of the European Society for Clinical Respiratory Physiology. 2002;20(5):1220–7. 10.1183/09031936.02.00022302 12449177

[5] Fireman E, Kraiem Z, Sade O, Greif J, Fireman Z. Induced sputum-retrieved matrix metalloproteinase 9 and tissue metalloproteinase inhibitor 1 in granulomatous diseases. Clinical and experimental immunology. 2002;130(2):331–7. 12390324 10.1046/j.1365-2249.2002.t01-1-02001.x PMC1906521

[6] Piotrowski WJ, Kiszałkiewicz J, Górski P, Antczak A, Górski W, Pastuszak-Lewandoska D, et al Immunoexpression of TGF-β/Smad and VEGF-A proteins in serum and BAL fluid of sarcoidosis patients. BMC Immunology. 2015;16(1):1–8. 10.1186/s12865-015-0123-y 26445225 PMC4595252

[7] Oshita Y, Koga T, Kamimura T, Matsuo K, Rikimaru T, Aizawa H. Increased circulating 92 kDa matrix metalloproteinase (MMP-9) activity in exacerbations of asthma. Thorax. 2003;58(9):757–60. 12947131 10.1136/thorax.58.9.757 PMC1746799

[8] Dancer RCA, Wood AM, Thickett DR. Metalloproteinases in idiopathic pulmonary fibrosis. European Respiratory Journal. 2011;38(6):1461–7. 10.1183/09031936.00024711 21700608

[9] Navratilova Z, Zatloukal J, Kriegova E, Kolek V, Petrek M. Simultaneous up-regulation of matrix metalloproteinases 1, 2, 3, 7, 8, 9 and tissue inhibitors of metalloproteinases 1, 4 in serum of patients with chronic obstructive pulmonary disease. Respirology. 2012;17(6):1006–12. Epub 2012/05/18. 10.1111/j.1440-1843.2012.02197.x . 22591289

[10] Kafasla P, Skliris A, Kontoyiannis DL. Post-transcriptional coordination of immunological responses by RNA-binding proteins. Nature immunology. 2014;15(6):492–502. 10.1038/ni.2884 24840980

[11] Gerstberger S, Hafner M, Tuschl T. A census of human RNA-binding proteins. Nat Rev Genet. 2014;15(12):829–45. 10.1038/nrg3813 25365966 PMC11148870

[12] Ivanov P, Anderson P. Post-transcriptional regulatory networks in immunity. Immunological reviews. 2013;253(1):253–72. Epub 2013/04/05. 10.1111/imr.12051 . 23550651 PMC6989036

[13] Gubin MM, Techasintana P, Magee JD, Dahm GM, Calaluce R, Martindale JL, et al Conditional knockout of the RNA-binding protein HuR in CD4(+) T cells reveals a gene dosage effect on cytokine production. Mol Med. 2014;20:93–108. 10.2119/molmed.2013.00127 24477678 PMC3960399

[14] Stellato C, Gubin MM, Magee JD, Fang X, Fan J, Tartar DM, et al Coordinate regulation of GATA-3 and Th2 cytokine gene expression by the RNA-binding protein HuR. J Immunol. 2011;187(1):441–9. 10.4049/jimmunol.1001881 21613615 PMC5801757

[15] Paschoud S, Dogar AM, Kuntz C, Grisoni-Neupert B, Richman L, Kuhn LC. Destabilization of interleukin-6 mRNA requires a putative RNA stem-loop structure, an AU-rich element, and the RNA-binding protein AUF1. Molecular and cellular biology. 2006;26(22):8228–41. 16954375 10.1128/MCB.01155-06 PMC1636780

[16] Chowdhury S, Dijkhuis A, Steiert S, Lutter R. IL-17 attenuates degradation of ARE-mRNAs by changing the cooperation between AU-binding proteins and microRNA16. PLoS genetics. 2013;9(9):26. 10.1371/journal.pgen.1003747 PMC3784493 24086143

[17] Abdelmohsen K, Gorospe M. RNA-binding protein nucleolin in disease. RNA biology. 2012;9(6):799–808. Epub 2012/05/24. 10.4161/rna.19718 22617883 PMC3495746

[18] Hirano K, Miki Y, Hirai Y, Sato R, Itoh T, Hayashi A, et al A multifunctional shuttling protein nucleolin is a macrophage receptor for apoptotic cells. The Journal of biological chemistry. 2005;280(47):39284–93. 16135517 10.1074/jbc.M505275200

[19] Liu W, Rosenberg GA, Liu KJ. AUF-1 mediates inhibition by nitric oxide of lipopolysaccharide-induced matrix metalloproteinase-9 expression in cultured astrocytes. J Neurosci Res. 2006;84(2):360–9. 16683234 10.1002/jnr.20895

[20] Fahling M, Steege A, Perlewitz A, Nafz B, Mrowka R, Persson PB, et al Role of nucleolin in posttranscriptional control of MMP-9 expression. Biochimica et biophysica acta. 2005;1731(1):32–40. Epub 2005/09/13. 10.1016/j.bbaexp.2005.08.005 . 16153722

[21] Akool el S, Kleinert H, Hamada FM, Abdelwahab MH, Forstermann U, Pfeilschifter J, et al Nitric oxide increases the decay of matrix metalloproteinase 9 mRNA by inhibiting the expression of mRNA-stabilizing factor HuR. Molecular and cellular biology. 2003;23(14):4901–16. Epub 2003/07/02. 12832476 10.1128/MCB.23.14.4901-4916.2003 PMC162218

[22] Mukherjee N, Corcoran DL, Nusbaum JD, Reid DW, Georgiev S, Hafner M, et al Integrative regulatory mapping indicates that the RNA-binding protein HuR couples pre-mRNA processing and mRNA stability. Molecular cell. 2011;43(3):327–39. Epub 2011/07/05. 10.1016/j.molcel.2011.06.007 21723170 PMC3220597

[23] Park MJ, Kim MS, Park IC, Kang HS, Yoo H, Park SH, et al PTEN suppresses hyaluronic acid-induced matrix metalloproteinase-9 expression in U87MG glioblastoma cells through focal adhesion kinase dephosphorylation. Cancer research. 2002;62(21):6318–22. Epub 2002/11/05. . 12414663

[24] Takahashi C, Sheng Z, Horan TP, Kitayama H, Maki M, Hitomi K, et al Regulation of matrix metalloproteinase-9 and inhibition of tumor invasion by the membrane-anchored glycoprotein RECK. Proceedings of the National Academy of Sciences of the United States of America. 1998;95(22):13221–6. Epub 1998/10/28. 9789069 10.1073/pnas.95.22.13221 PMC23764

[25] Yoon JH, De S, Srikantan S, Abdelmohsen K, Grammatikakis I, Kim J, et al PAR-CLIP analysis uncovers AUF1 impact on target RNA fate and genome integrity. Nat Commun. 2014;5(5248). 10.1038/ncomms6248 PMC4291169 25366541

[26] Rabe KF, Hurd S, Anzueto A, Barnes PJ, Buist SA, Calverley P, et al Global strategy for the diagnosis, management, and prevention of chronic obstructive pulmonary disease—GOLD executive summary. Am J Resp Crit Care. 2007;176(6):532–55. 10.1164/rccm.200703-456SO . 17507545

[27] Global Initiative for Asthma. Medicine on the Net. 2007;13(9):14-.

[28] Katzenstein AL, Myers JL. Nonspecific interstitial pneumonia and the other idiopathic interstitial pneumonias: classification and diagnostic criteria. The American journal of surgical pathology. 2000;24(1):1–3. Epub 2000/01/13. . 10632482 10.1097/00000478-200001000-00001

[29] American Thoracic Society/European Respiratory Society International Multidisciplinary Consensus Classification of the Idiopathic Interstitial Pneumonias. This joint statement of the American Thoracic Society (ATS), and the European Respiratory Society (ERS) was adopted by the ATS board of directors, June 2001 and by the ERS Executive Committee, June 2001. Am J Respir Crit Care Med. 2002;165(2):277–304. Epub 2002/01/16. 10.1164/ajrccm.165.2.ats01 . 11790668

[30] Travis WD, Costabel U, Hansell DM, King TE Jr., Lynch DA, Nicholson AG, et al An official American Thoracic Society/European Respiratory Society statement: Update of the international multidisciplinary classification of the idiopathic interstitial pneumonias. Am J Respir Crit Care Med. 2013;188(6):733–48. Epub 2013/09/17. 10.1164/rccm.201308-1483ST . 24032382 PMC5803655

[31] Petrek M, Gibejova A, Drabek J, Mrazek F, Kolek V, Weigl E, et al CC chemokine receptor 5 (CCR5) mRNA expression in pulmonary sarcoidosis. Immunol Lett. 2002;80(3):189–93. 10.1016/s0165-2478(01)00324-8 . 11803051

[32] Kriegova E, Arakelyan A, Fillerova R, Zatloukal J, Mrazek F, Navratilova Z, et al PSMB2 and RPL32 are suitable denominators to normalize gene expression profiles in bronchoalveolar cells. BMC Mol Biol. 2008;9(69):1471–2199. 10.1186/1471-2199-9-69 PMC2529339 18671841

[33] Hudy MH, Proud D. Cigarette smoke enhances human rhinovirus-induced CXCL8 production via HuR-mediated mRNA stabilization in human airway epithelial cells. Respiratory research. 2013;14:88 Epub 2013/08/31. 10.1186/1465-9921-14-88 23988199 PMC3848374

[34] Glader P, Moller S, Lilja J, Wieslander E, Lofdahl CG, von Wachenfeldt K. Cigarette smoke extract modulates respiratory defence mechanisms through effects on T-cells and airway epithelial cells. Respiratory medicine. 2006;100(5):818–27. 16242311 10.1016/j.rmed.2005.09.008

[35] Grunewald J, Eklund A. Role of CD4+ T cells in sarcoidosis. Proceedings of the American Thoracic Society. 2007;4(5):461–4. 17684290 10.1513/pats.200606-130MS PMC2647597

[36] Kechavarzi B, Janga SC. Dissecting the expression landscape of RNA-binding proteins in human cancers. Genome biology. 2014;15(1):R14 Epub 2014/01/15. 10.1186/gb-2014-15-1-r14 24410894 PMC4053825

[37] Techasintana P, Davis JW, Gubin MM, Magee JD, Atasoy U. Transcriptomic-Wide Discovery of Direct and Indirect HuR RNA Targets in Activated CD4+ T Cells. PloS one. 2015;10(7). 10.1371/journal.pone.0129321 PMC4498740 26162078

[38] Raghavan A, Ogilvie RL, Reilly C, Abelson ML, Raghavan S, Vasdewani J, et al Genome-wide analysis of mRNA decay in resting and activated primary human T lymphocytes. Nucleic acids research. 2002;30(24):5529–38. 12490721 10.1093/nar/gkf682 PMC140061

[39] Vestbo J, Hurd SS, Agusti AG, Jones PW, Vogelmeier C, Anzueto A, et al Global strategy for the diagnosis, management, and prevention of chronic obstructive pulmonary disease: GOLD executive summary. Am J Respir Crit Care Med. 2013;187(4):347–65. Epub 2012/08/11. 10.1164/rccm.201204-0596PP . 22878278

[40] Skjot-Arkil H, Clausen RE, Nguyen QH, Wang Y, Zheng Q, Martinez FJ, et al Measurement of MMP-9 and -12 degraded elastin (ELM) provides unique information on lung tissue degradation. BMC pulmonary medicine. 2012;12(1):34 Epub 2012/07/24. 10.1186/1471-2466-12-34 . 22818364 PMC3515477

[41] Paulissen G, Rocks N, Quesada-Calvo F, Gosset P, Foidart JM, Noel A, et al Expression of ADAMs and their inhibitors in sputum from patients with asthma. Mol Med. 2006;12(7–8):171–9. Epub 2006/11/08. 10.2119/2006-00028.Paulissen 17088949 PMC1626598

