# Supplementary material for: Expression Profile of Six RNA-Binding Proteins in Pulmonary Sarcoidosis
Source: PLoS One. 2016 Aug 30;11(8):e0161669. doi: 10.1371/journal.pone.0161669 (PMC5004853; doi:10.1371/journal.pone.0161669)
Supplement: S1 File — (DOC) [file pone.0161669.s001.doc]

**Methods**

**1.Subjects**

In the time of BAL (blood) sample collection, 33(4) pulmonary S and 15 IIPs patients had systemic/oral steroid therapy and 21 COPD and 15 asthmatic patients were treated by inhaled steroid. Only COPD/asthmatic/IIPs individuals without history of acute exacerbation 6 months prior/after the study were enrolled. None of the control subjects had ever experienced chronic respiratory symptoms and their BAL had normal cellular profile (Table S1). The BAL (blood) samples were obtained from S/healthy control/COPD/asthmatic/IIPs patients who were never smokers (35(6)/13/8/9/9), ex-smokers for at least 2 years (11(3)/6/14/6/4) and current smokers (2(0)/4/8/3/4). The smoking status was not available in 2/0/0/1/2 subject(s) with pulmonary S/healthy control/COPD/asthma/IIPs.

**2.Results**

**2.1.BA expression of RBPs mRNAs in chronic obstructive pulmonary disease (COPD)**

Although Dunn's Multiple Comparison of five groups showed similar expressions of all investigated genes, Mann-Whitney test showed the relative expressions of three RBPs (AUF1, HuR and NCL) and RECK to be significantly decreased in COPD patients than those in healthy controls (*p*<0.01, *p*<0.05, *p*<0.05 and *p*<0.01; S4 Table). These down-regulated expressions (*p*_AUF1_<0.05, *p*_HuR_<0.01, *p*_NCL_<0.01 and *p*_RECK_<0.05; S4 Table) remained significant in the sub-analysis of current and ex-smokers (without never smokers). The relative expressions of all investigated genes did not differ between COPD and asthmatic patients (*p*>0.05; S4 Table).

**2.2.BA expression of RBP mRNA in asthma**

Mann-Whitney test showed the relative expressions of AUF1 and RECK to be decreased in asthmatic patients than those in healthy controls (*p*<0.05, *p*<0.05; S4 Table). However, the relative expression of all investigated genes did not differ between asthmatic never smokers and healthy never smokers (for all comparisons *p*>0.05; S4 Table).

**2.3.BA expression of RBP mRNA in idiopathic interstitial pneumonia (IIPs)**

Dunn's Multiple Comparison of five groups including all recruited patients regardless of smoking status showed significantly decreased relative expressions of all RBPs (AUF1, HuR, TIA, TIAR, NCL and PCBP2) and RECK in our patients with IIPs compared to those in healthy controls (for all *p*<0.001, S4 Table). The subsequent sub-analysis of never smokers showed that AUF1 (*p_healthy controls_*<0.001, *p_COPD_*<0.01, *p_asthma_*<0.05), TIA (*p*<0.001*_healthy controls_*, *p_COPD_*<0.01, *p_asthma_*<0.05), NCL (*p*<0.001*_healthy controls_*, *p_COPD_*<0.001, *p_asthma_*<0.01), PCBP2 (*p*<0.001*_healthy controls_*, *p_COPD_*<0.05, *p_asthma_*<0.05) and RECK (*p_healthy controls_*<0.001, *p_COPD_*<0.05, *p_asthma_*<0.05) remained down-regulated in IIPs compared to healthy controls, COPD and asthmatic patients (S4 Table).

**2.4.Correlation analyses**

Among COPD patients, RECK decreased in parallel with decreasing expression of AUF1 (*p*<0.001), NCL (*p*<0.001), TIA (*p*<0.001) and PCBP2 (*p*<0.001). Among asthmatic patients, RECK decreased in parallel with decreasing expression of AUF1 (*p*<0.001), NCL (*p*=0.006), HuR (*p*=0.005) and PCBP2 (*p*=0.01). Among our patients with IIPs, RECK decreased in parallel with decreasing expression of AUF1 (*p*=0.005), NCL (*p*=0.03) and HuR (*p*<0.03). By contrast to all patient groups (S/COPD/asthma/IIPs), these positive correlations were not observed in healthy controls (*p*>0.05). There were not any relationships between PTEN and RBPs in controls groups of our healthy controls or patients with COPD, asthma and IIPs.

Regarding the cellular profile of BAL in our disease control groups, the increasing relative expression of NCL was associated with decreasing absolute and relative numbers of eosinophils (*p*=0.01 and *p*=0.002) in COPD group.

**2.5.The effect of smoking and age on BA expression of RBPs mRNAs**

The relative mRNA expressions didn’t differ between never smokers and cigarette smokers including ex-smokers and current smokers in any control group (healthy controls, COPD, asthma and IIPs) (*p*>0.05). There was also no difference between never and current smokers among COPD patients (*p*>0.05).

Regarding age, neither RBPs nor the inhibitors differ between our young (*≤* 40 years, n=12) and elderly (>40 years, n=11) (*p*>0.05) in healthy control group. The relative expressions didn’t differ between young (*≤* 65 years, n=17) and elderly (>65 years, n=13) COPD patients (*p*>0.05) and between our young (*≤* 45 years, n=10) and elderly (>45 years, n=9) patients with asthma (*p*>0.05). Similarly, the relative expressions didn’t differ between our young (*≤* 55 years, n=9) and elderly (>55 years, n=10) patients with IIPs (*p*>0.05).
