# Supplementary material for: Expression Profile of Six RNA-Binding Proteins in Pulmonary Sarcoidosis
Source: PLoS One. 2016 Aug 30;11(8):e0161669. doi: 10.1371/journal.pone.0161669 (PMC5004853; doi:10.1371/journal.pone.0161669)
Supplement: S1 Table — (DOC) [file pone.0161669.s002.doc]

| **pulmonary disease** | **percentile** | **total number of BAL cells** | **Macrophages** | **Lymphocyts** | **Neutrophils** | **Eosinophils** | **CD3** | **CD4** | **CD8** | **CD4/CD8** | **CD19** |
| --- | --- | --- | --- | --- | --- | --- | --- | --- | --- | --- | --- |
|  |  | **millions per 1ml** | **%** | **%** | **%** | **%** | **%** | **%** | **%** | **%** | **%** |
| **C (n=23)** | 25th | 0.6 | 90.1 | 2.5 | 0.7 | 0.4 | 68.4 | 32.3 | 26.3 | 1.0 | 0.0 |
|  | **median** | **0.8** | **94.2** | **5.3** | **1.3** | **0.5** | **81.5** | **41.5** | **32.0** | **1.3** | **1.0** |
|  | 75th | 1.1 | 95.8 | 7.0 | 3.0 | 0.5 | 89.0 | 48.8 | 39.8 | 2.0 | 1.0 |
| **S (n=50)** | 25th | 0.46 | 64.0 | 16.0 | 0.0 | 0.0 | 82.8 | 47.3 | 9.8 | 2.7 | 0.0 |
|  | **median** | **0.68** | **72.5** | **24.0** | **2.0** | **0.0** | **89.0** | **71.5** | **13.0** | **5.7** | **0.0** |
|  | 75th | 0.99 | 78.0 | 31.0 | 4.0 | 1.0 | 92.0 | 78.3 | 17.3 | 7.3 | 1.0 |
| **COPD (n=30)** | 25th | 0.5 | 72.0 | 0.5 | 1.2 | 0.0 | 42.0 | 18.0 | 10.0 | 0.9 | 0.0 |
|  | **median** | **0.9** | **85.4** | **2.5** | **3.8** | **0.3** | **74.0** | **34.0** | **17.0** | **1.5** | **0.0** |
|  | 75th | 1.9 | 93.0 | 11.7 | 5.7 | 1.3 | 79.0 | 45.0 | 38.0 | 4.2 | 0.0 |
| **Asthma (n=19)** | 25th | 0.5 | 76.8 | 2.0 | 0.6 | 0.4 | 45.5 | 27.5 | 13.0 | 0.8 | 0.0 |
|  | **median** | **0.6** | **84.0** | **6.0** | **1.5** | **2.0** | **68.5** | **33.0** | **20.5** | **1.9** | **0.1** |
|  | 75th | 1.2 | 94. | 12.5 | 3.8 | 8.1 | 84.0 | 53.5 | 28.0 | 3.7 | 1.0 |
| **IIPs**  **(n=19)** | 25th | 1.1 | 74.6 | 6.9 | 1.8 | 0.1 | 66.0 | 17.5 | 20.0 | 0.6 | 0.0 |
|  | **median** | **1.5** | **82.3** | **8.5** | **2.5** | **0.8** | **74.0** | **34.0** | **24.0** | **1.3** | **0.0** |
|  | 75th | 2.4 | 89.6 | 14.6 | 5.8 | 4.5 | 90.0 | 55.5 | 41.5 | 3.0 | 1.0 |

S1 Table. BAL cellular profiles of the investigated groups.

Legend: C, healthy Controls; S, pulmonary Sarcoidosis; COPD, Chronic Obstructive Pulmonary Disease; IIPs, Idiopathic Interstitial Pneumonias.
