# Supplementary material for: Expression Profile of Six RNA-Binding Proteins in Pulmonary Sarcoidosis
Source: PLoS One. 2016 Aug 30;11(8):e0161669. doi: 10.1371/journal.pone.0161669 (PMC5004853; doi:10.1371/journal.pone.0161669)
Supplement: S2 Table — (DOC) [file pone.0161669.s003.doc]

S2 Table. Prime sequences for RNA-binding proteins, two inhibitors of proteolytic activity and a housekeeping gene.

|  | Gene abbreviation | Gene name | General function | GenBank accession number | Forward and reverse primers | LNA probe* |
| --- | --- | --- | --- | --- | --- | --- |
| RBPs | AUF1 | Human AU-rich element RNA-binding protein AUF1 mRNA, complete cds | ARE-mRNA destabilization | U02019.1 | gcgaagattgacgccagta | #88 |
|  |  |  |  |  | tcccagctaaggcctcctat |  |
|  | HuR | Human HuR RNA binding protein (HuR) mRNA | ARE-mRNA stabilization | U38175.1 | ccaggcgcagagattcag | #72 |
|  |  |  |  |  | ggttgtagatgaaaatgcaccag |  |
|  | NCL | Homo sapiens nucleolin | Ribosomal RNA biosynthesis | NM_005381.2 | ccacttgtccgcttcaca | #70 |
|  |  |  |  |  | tcttggggtcaccttgattt |  |
|  | TIA | Human polyadenylate binding protein | Inhibition of translation | M77142.1 | agggagctggacctggag | #46 |
|  |  |  |  |  | tggaaaggttaccgacgtataga |  |
|  | TIAR | GDB Homo sapiens nucleolysin TIAR mRNA |  | M96954.1 | ggcaaccatggaatcaaca | #84 |
|  |  |  |  |  | agcaccaaatccacccatc |  |
|  | PCBP2 | Homo sapiens poly(rC) binding protein 2 | replication or translation of many RNA viruses | NM_005016.5 | agatctgcgtggtcatgttg | #14 |
|  |  |  |  |  | tgctgtacctgtcctgacca |  |
| Inhibitors of proteolytic activity | RECK | Homo sapiens reversion-inducing-cysteine-rich protein with kazal motifs | Inhibition of MMP secretion and induction of its inhibitors | NM_021111.2 | caagtgtccttcgctcttgg | #42 |
|  |  |  |  |  | cacataatgggcaacaagca |  |
|  | PTEN | Homo sapiens phosphatase and tensin homolog | Inhibition of MMP secretion and induction of its inhibitors | NM_000314.4 | gcacaagaggccctagatttc | #60 |
|  |  |  |  |  | cgcctctgactgggaatagt |  |
| Housekeeping gene | PSMB2 | Homo sapiens proteasome (prosome, macropain) subunit, beta type, 2 | Housekeeping gene | NM_002794.4 | gtgagagggcagtggaactc | #50 |
|  |  |  |  |  | gaaggttggcagattcagga |  |

Legend: Legend: ARE, Adenine and uracil-Rich Element; MMP, matrix metalloproteinase, *Number of Locked Nucleic Acid (LNA) probe associated with a prime pair according to http://universalprobelibrary.com

References

1. Ivanov P, Anderson P (2013) Post-transcriptional regulatory networks in immunity. Immunol Rev 253: 253-272.

2. Chowdhury S, Dijkhuis A, Steiert S, Lutter R (2013) IL-17 attenuates degradation of ARE-mRNAs by changing the cooperation between AU-binding proteins and microRNA16. PLoS Genet 9: 26.

3. Stellato C, Gubin MM, Magee JD, Fang X, Fan J, et al. (2011) Coordinate regulation of GATA-3 and Th2 cytokine gene expression by the RNA-binding protein HuR. J Immunol 187: 441-449.

4. Abdelmohsen K, Gorospe M (2012) RNA-binding protein nucleolin in disease. RNA Biol 9: 799-808.

5. Zhang T, Kruys V, Huez G, Gueydan C (2002) AU-rich element-mediated translational control: complexity and multiple activities of trans-activating factors. Biochem Soc Trans 30: 952-958.

6. Reyes R, Alcalde J, Izquierdo JM (2009) Depletion of T-cell intracellular antigen proteins promotes cell proliferation. Genome Biol 10: 2009-2010.

7. Takahashi C, Sheng Z, Horan TP, Kitayama H, Maki M, et al. (1998) Regulation of matrix metalloproteinase-9 and inhibition of tumor invasion by the membrane-anchored glycoprotein RECK. Proc Natl Acad Sci U S A 95: 13221-13226.

8. Park MJ, Kim MS, Park IC, Kang HS, Yoo H, et al. (2002) PTEN suppresses hyaluronic acid-induced matrix metalloproteinase-9 expression in U87MG glioblastoma cells through focal adhesion kinase dephosphorylation. Cancer Res 62: 6318-6322.

9. Kriegova E, Arakelyan A, Fillerova R, Zatloukal J, Mrazek F, et al. (2008) PSMB2 and RPL32 are suitable denominators to normalize gene expression profiles in bronchoalveolar cells. BMC Mol Biol 9: 1471-2199.
