# Supplementary material for: Expression Profile of Six RNA-Binding Proteins in Pulmonary Sarcoidosis
Source: PLoS One. 2016 Aug 30;11(8):e0161669. doi: 10.1371/journal.pone.0161669 (PMC5004853; doi:10.1371/journal.pone.0161669)
Supplement: S3 Table — (DOC) [file pone.0161669.s004.doc]

S3 Table. mRNA expression for RNA-binding proteins (RBPs) and one inhibitor of proteolytic activity (RECK) in the patients with pulmonary sarcoidosis compared to 4 control groups including healthy individuals and the patients with COPD, asthma and IIPs.

| (n) | Pulmonary sarcoidosis (50) | Healthy controls (23) | COPD (30) | Asthma (19) | IIPs (19) |
| --- | --- | --- | --- | --- | --- |
| AUF1 | 0.34 (0.15; 0.58) | 1.89 (1.24; 2.13)***### | 1.16 (0.83; 1.92)*** | 1.31 (0.38; 1.92)** | 0.15 (0.04; 0.29) ns |
| HuR | 0.24 (0.02; 0.57) | 0.93 (0.6; 1.49)*** | 0.46 (0.15; 1.06)ns | 0.76 (0.22; 1.01)* | 0.15 (0.02; 0.25) ns |
| NCL | 0.33 (0.16; 0.66) | 2.24 (1.75; 2.94)***## | 1.71 (1.03; 2.39)***# | 2.36 (0.97; 3.47)*** | 0.14 (0.02; 0.29) ns |
| TIA | 0.14 (0.01; 0.31) | 0.83 (0.63; 1.04)***### | 0.62 (0.44; 0.93)***# | 0.81 (0.31; 1.16)***# | 0.06 (0.03; 0.14) ns |
| TIAR | 0.92 (0.47; 1.20) | 1.24 (1.05; 1.54)* | 1.2 (0.92; 1.44) ns | 1.35 (0.98; 1.83) ns | 0.36 (0.19; 0.57)* |
| PCBP2 | 0.19 (0.01; 0.74) | 0.58 (0.36; 1.78) ns | 0.38 (0.18; 0.82) ns | 0.57 (0.01; 1.23) ns | 0.01 (0.01; 0.02)**# |
| RECK | 0.02 (0.01; 0.09) | 0.12 (0.10; 0.16)***# | 0.07 (0.05; 0.13) ns | 0.09 (0.05; 0.13)* | 0.01 (0.01; 0.02) ns |

Legend: The relative mRNA expressions are presented as median with 25^th^ and 75^th^ percentiles in bracket; COPD, chronic obstructive pulmonary disease; IIPs, idiopathic interstitial pneumonia; ns, not significant.

Dunn's Multiple Comparison Test:

Multiple comparison regardless of smoking status****p*<0.001,***p*<0.01,**p*<0.05;

Multiple comparison of never smokers ###*p*<0.001, ##*p*<0.01, #*p*<0.05
