# Supplementary material for: Expression Profile of Six RNA-Binding Proteins in Pulmonary Sarcoidosis
Source: PLoS One. 2016 Aug 30;11(8):e0161669. doi: 10.1371/journal.pone.0161669 (PMC5004853; doi:10.1371/journal.pone.0161669)
Supplement: S4 Table — (DOC) [file pone.0161669.s005.doc]

S4 Table. Comparisons of mRNA expression for RNA-binding proteins (RBPs) and one inhibitor of proteolytic activity (RECK) between 4 control groups including the healthy individuals and the patients with COPD, asthma and IIPs .

| (n) | COPD(30) vs | | | Asthma(19) vs | | IIPs(19) vs |
| --- | --- | --- | --- | --- | --- | --- |
| (n) | healthy controls (23) | Asthma (19) | IIPs (19) | healthy controls (23) | IIPs (19) | healthy controls (23) |
| AUF1 | ↑++♦ | ns | ↓***## | ↑+ | ↓***# | ↑***### |
| HuR | ↑+♦♦ | ns | ↓* | ns | ↓** | ↑*** |
| NCL | ↑+♦♦ | ns | ↓***### | ns | ↓***## | ↑***### |
| TIA | ↑♦ | ns | ↓***## | ns | ↓***# | ↑***### |
| TIAR | ns | ns | ↓*** | ns | ↓***# | ↑***# |
| PCBP2 | ns | ns | ↓***# | ns | ↓***# | ↑***### |
| RECK | ↑++♦ | ns | ↓***# | ↑+ | ↓***# | ↑***### |

Legend: ↑increased mRNA expressions; ↓decreased mRNA expressions; COPD, chronic obstructive pulmonary disease; IIPs, idiopathic interstitial pneumonia; ns, not significant.

Mann-Whitney test U test:

regardless of smoking status ++ p<0.01, + p<0.05,

current and ex-smokers ♦♦p<0.01, ♦p<0.05.

Dunn's Multiple Comparison Test:

Multiple comparison regardless of smoking status***p<0.001,**p<0.01,*p<0.05;

Multiple comparison never smokers ###p<0.001, ##p<0.01, #p<0.05.
